# 

**Published:** 1920-04-03

**Authors:** 


Supplement to "The Hospital," October 16, 1920.

### THE HOSPITAL

*THE MODERN NEWSPAPER OF ADMINISTRATIVE MEDICINE AND INSTITUTIONAL LIFE*

INCLUDING

ADMINISTRATION, NATIONAL INSURANCE, HEALTH

AND

THE INTERESTS OF ALL INSTITUTIONAL WORKERS

LIBRARY

OCT 27 1920

Surgeon General's Office

APRIL TO SEPTEMBER 1920

VOLUME LXVIII

SURGEON GENERAL'S

241421

LIBRARY

LONDON
PUBLISHED BY THE SCIENTIFIC PRESS (LIMITED), AT *THE HOSPITAL* BUILDING
28 & 29 SOUTHAMPTON STREET, STRAND, W.C. 2.
MDCCCCXX

W1
NO 69X

### INDEX TO VOL. LXVIII.

Aberystwyth Infirmary, 648, Sept. 25, vi
Action Against Ealing Medical Officer, 466
Acton, Mr. Frederick, 438
Actors' Orphanage, Apri1 17, vi, 100
Admiralty, 161
Adopted Children and State Control, 412
Aftermath of War, The, 461
Agricultural Contributions, 300
Aikins, Miss C., 418
Air Mails, 634
Airmen, Civilian, Medical Examination of, 480
Alcohol, Hospitals and Budget Increase, 106
Aldershot, Cambridge Hospital, 231
Alexandra Day, 106, 364, Supp., Sept. 25
Allbutt, Sir Clifford, 375, 401
Allenby, Lord, 497, 532
Almoners under the Poor Law, 444
Altar of Domestic Dirt, The, 507
Ambulance Train for Poultry Demonstration, 152
American Army Nurse Corps, 533
American College of Surgeons, 447
American Hospital Association, The, 353
Anæmia as a True Disease, 640
Anæsthetic, The New, 59
Anæsthetists, Nurses as, 292
Analysis Journal, The, Supp., Aug. 14
Animal Asthmas, 498
Annual Meetings, 48, 76
Anthrax Prevention, 79; Shaving Brushes, 242; Fight Against, 295
Appel, Mrs. Ellen, 94
**APPOINTMENTS, MEDICAL:** 26, 200, 212, 287, Aug. 28, vi
**APPOINTMENTS: MATRONS AND SISTERS,** April 10, vi, April 17, vi, 102, 128, 158, May 15, iv, 212, 238, 262, June 12, vi, 312, July 10, vi, 422, 444, 471, 494, 516, 538, August 28, vi, Sept. 4, xx, 612, Sept. 18, vi, Sept. 25, vi
Army Nurses, 40, 118, 257, 370, 490, Sept. 4, xx, 608, 609, 626
Arsenic in Sugar, 54
Artificial Limbs, 422
Art of Healing, The, 421
Ascot Week and Hospitals, 267
As Others See Us, 38
Association of Hospital Matrons, 307, 332
Association of Poor-Law Unions, 537
Association of Surgeons, 3
Astor, Lady, 392, 490, 555
Asylum War Hospitals, 623
Athlone, Earl of, 189
Attainment, The Way of, 534
Australia: Public Health Administration, 25; Notes, 75, 630; League of Mercy, 106; War Pensions, 287; Private Hospitals, 403; Hospital Questions, 558; Bush Nursing Association, 610

Baby Week (*see* Infant Welfare)
Bangor City Council, 268
Bann, Miss E. M., 41
Barker, Mr. H. A., 377
Barling, Sir Gilbert, 293
Barnes Guardians, 4
Barnet, Wellhouse Hospital, 543
Barry, The Late Sir John Wolfe, 189
Barton, Miss E. C., 175
Bath, Tropical Diseases Hospital, 398
Batten, Mrs., 491
Bedford, Adeline, Duchess of, 92, 98
Belfast: Organisation of Subscriptions, 451; Guardians and Nurse-Training, 555
Belloc, Mr. Hilaire, 485
Bentinck, Lady Charles, 521
Berkshire Hospitals, Pageant for, 365
Bermondsey Fiasco, A, 133
Bermondsey: Infirmary, 308, 392, 417, Borough Council, 354, 365
Bio-Chemistry, A Chart in, 292
Birmingham: Hospital Saturday, 378; District Nursing Association, 490; Joint Hospital Appeal, 633
Birth Control, 352, 460
Birth-Rate: Sex Education, 45; Returns, 208
Bittern Park Nursing Association, 648
Bland-Sutton, Sir J., 241
Blankets, The Care of, 287
Blind, The, 119, 120; Blinded Soldiers, 128; Pensions, 132; Advisory Committee's Report, 543, 641; Poor-Law Blind, 543; Training, 641
Blood Pressure and Fatigue, 429
Bone and Cartilage, Grafting of, 109

**BOOK WORLD:**
Aitchison: A Medical Handbook for the Use of Practitioners and Students, 9
Balfour: War Against Tropical Disease, 318
Barber: A British Nurse in Bolshevik Russia, 527
Bardswell: Advice to Consumptives, 358
Barton: The Nursing of Chronic Patients, 65
Bayly: Venereal Diseases, 484
Bear: British System of Physical Education, 250
Berkeley: A Handbook of Midwifery, 650
Berkeley and Bonney: A Text-Book of Gynæcological Surgery, 9
Blackham: Primer of Tropical Hygiene, 250; Military Sanitation, 602
Cabot: Modern Urology, 398
Cammidge: Dietetic Dietary and Cookery, Sept. 4, xix
Collis: The Industrial Clinic, 358
Davies: Ward Tales, 483

Book World—*continued.*
Davis: Obstetric and Gynæcological Nursing, 10
Donald: An Introduction to Midwifery, 602
Elmslie: The After-Treatment of Wounds and Injuries, 527
Findlay: Syphilis in Children, 114
Fletcher: The Link Between Practitioner and Laboratory, 249
Fox: Care of Sane Epileptic Children, 250
Friel: Electric Ionisation, 527
Gibb: The Realities of War, 357
Glaister: Manual of Hygiene for Students, 602
Gough: Half-past Twelve, 250
Grant: The Diary of a Police Surgeon, Sept. 4, xix
Hardyman: A Challenge, 10
Hart: The Doctor's Manual, 358
Hill: Sanitation for Public Health Nurses, 484
Holt: The Diseases of Infancy and Childhood, 527
Hutchinson: On Facial Neuralgia and Its Treatment, 9
James: Malaria at Home and Abroad, 358
Jones: Castellinaris, 10
Jones: Chemistry for Public Health Students, 9
Jones: Diversions in Sicily, 10
Keith: Engines of the Human Body, 9
Lang: A Handbook to British Mosquitoes, 484
Leftwich: Index of Symptoms, 250
McCann: The Science of Eating, 484
Macdowall: Training of Mentally Defective Children, 650
McMonach: Venereal Diseases, Sept. 4, xix
Martindale and Westcott: The Extra Pharmacopœia, 602
Masterman: The Essentials of Tropical Medicine, 602
Miller: Functional Nerve Disease, 249
Neuman: All About Baby, 484
Ostrom: Massage and the Original Swedish Movements, 10
Overend: Radiography of the Chest, 249
Porter: Diseases of the Throat, Nose, and Ear, 114
Robinson: Cunningham's Practical Anatomy, 114
Roget: Altitude and Health, 358
Ross: Handbook of Anæsthetics, 9
Russ: The Conquest of Venereal Disease, 458
Saintsbury: Notes on a Cellar-Book, 483
Scharlieb: The Welfare of the Expectant Mother, 114; Changes of Life, 114

BOOK WORLD—*continued.*
Short: The New Physiology in Surgical and General Practice, Sept. 4, xix
Slemons: The Nutrition of the Fœtus, 602
Snowman: Lenzmann's Manual of Emergencies, 113
Spencer: Animal Experiments and Surgery, 483
Thomson: Tuberculosis and Public Health, 249
Transactions of the Incorporated Sanitary Association of Scotland, 1919, 484
Treloar: A Lord Mayor's Diary, 357
Wallace: Child Welfare, 483
Westland: The Wife and Mother, 484
White: An Atlas of Syphilis, 84
Williams: Minor Surgery and Bandaging, 250
Wright: Industrial Nursing, 527
Bournemouth and British Dental Association, 522
Boy Labour in Mines, 120
Boy Scouts, 505
Bradford, Miss Alice, 26
Bradford: Municipal Hospital, 32, 128; Health Committee, 257
Bridlington Red Cross Convalescent Home, 628
Brighton: Cinema to Help Hospitals, 638; Ambulance Services, 646
Bristol: Proposed Hospital Amalgamation, 4, 164
British Hospitals Association, 159, 162, 189, 292, 293, 310, 315, 352, 367, 448, 652
British Medical Association, 315; Meeting at Cambridge, 351, 360, 375, 376, 381
British Pharmaceutical Conference, 288, 514
British Science Guild, 292, 309, 430
Brussels Conference, 277
Buchan, Dr. J. J., 233
Budget, The, 113
Bunyan: Was He Mad? 6
Burdett, Sir Henry, Death of, 129, 139, 161, 169, 181, 198, 229, 391, 557
Burnett, Sir Napier, 199, 236, 256, 291, 326, 373, 383, 408, 475
Business Houses and the Hospitals, 592

Cairo: Suggested British Hospital, 497, 532
"Calendar" System for Raising Money, The, Supplement, August 21
Camberwell: Infirmary, 6, 67, 460; Board of Guardians, 67, 281
Canada: Ontario Hospitals, 93; Nurse-Training Schools, 510
Cancer: A Crusade, 35; Research, 104; Experimental Studies, 451, 479
Cann, Miss, 393
Canterbury Cricket Week, 478
Cape Hospital Board, 537
Capetown University, 594
Capital of the Central Funds, The, 495
Capitation Grant Fallacy, A, 539
Cardiac Efficiency, 7
Cardiff: Mental Hospital, 308, 449, 519; Order of St. John, 466; Prince of Wales's Hospital for Limbless Men, 537
Carley, Miss Bessie, 175
Carmarthenshire: Isolation Hospital, 107; Public Health Committee, 465
Cavell, Edith: Statue, 15, 92; French Monument, 307, 311; Italian Tribute, 307; Homes of Rest, 308, 610
Census, The, 67, 523
Central Association for the Care of the Mentally Defective, 594
Central Committee for the Care of Cripples, 155, 157
Central Hospital Board for London, A, 159, 633
Central Midwives Board, 15, 258, 393
Chaplains, Hospital, 210
Charity Commissioners, 476
Charity Lands, Sale of, 370
Chartered Society of Massage (*see* Incorporated Society of Trained Masseuses)
Chertsey Nursing Association, 175
Cheshire County Nursing Association, 533
Chesterfield Municipal Maternity Hospital, 133
Chicken Pox at Glasgow, 158
Children and Strong Drink, 529
Children's Courts, 217
Children's Fears, 43
Children's Jewel Fund, 207
Child Welfare (*see* Infant Welfare)
China, Hospitals in, 511
Chinese Hospital, Life in a, 651
Choice Before Us, The, 289
Cholera in Korea, 560
Cinema, The: Clean Milk Film, 71; Ventilation, 191
City Churches, 210
City Coroner's Annual Report, The, 36
Civic Education League, 550
Civil Service Salary Award, The, 426
Clarke, the Late Mr. J. G., 450
Cleansing of Cups, The, 101
*Clinical Journal, The,* 426
Club for Nursing Sisters, 365
Coal Strike, The, 609, 614
Colchester: District Nursing Association, 16; Board of Guardians, 40
Coleman, Dr. C. J., 302, 303
College of Ambulance, 281
**COLLEGE OF NURSING** (*see also* State Registration of Nurses), 14, 205, 276, 305, 329, 437
Annual Report, 174, 183
Annual Meeting, 280, 333, June 12, vi
Badges, April 10, vi, 91, May 15, iv, 238
Registration Fee, The, 438
The College Bulletin, May 15, iv, 416, 531
Nursing Conference, 280, June 12, vi, 306, 330, 371
Hours of Employment Bill, 256, 262, 352, 364
The Elections, 38, 204
Information and Inquiry Bureau, 534
Nurses' Hours, 39
Residential Club, 626
Studentships, 174, 175, May 15, iv, 306
Scottish Board, 184, 471
Irish Board, 185, 494
CENTRES:
Birmingham, 102, 128, 238, 312, 336, 394, 444
Brighton, 158, 312, 368, Sept. 18, vi
Durham, June 12, vi, 494
Edinburgh, 102, 128, 471
Leeds, 26, 494
Liverpool, 102, 158
London, 153, 238, 438, 444, 554
Manchester, 158
Colloids in Medical Practice, 269
Committeeman's Confession, A, 266
Condensed Milk, 606, 615, 619
Conference Habit, The, 305
Connaught, The Duke of, 291
Contributions from Patients, 2
Convalescence, The Art of, 201
Cookery and Food Exhibition, 647
Copeland, Col. A. J., 132
Cornwall County Nursing Association, 176
Cost of Living, 66
Cottle, Dr. E. W., The Late, 30
Cotton Trade, Children in the, 18
Council for Promoting the Higher Training of Midwives, 186
County Nursing Schemes, Supp., May 8
Cow, What It Means to Keep a, 294
Cricket Day for Hospitals, 616
Crisis, The Hospital, 27
Croydon Mental Hospital, 427
Crumpsall Infirmary, 282
Cup-Tie Programme, The, 105
Cutler, Miss Beatrice, 92
Cutting, Mr. P., 634

*Daily Telegraph* Fund for Nurses, 14, 40, 66, 92, 437
Dale, Mr. G. B., July 10, vi
Dalgleish, Col. R., 497
"Damnable," 43
Dash, Mr. George, 82
Davies, Dr. Morgan, 624
Dees, Mr. J. A., 31, 108
De La Rue & Co.'s Medical Service, 352, 405, 441, 467
Dempsey, Sir A., 447
Dental Conference, 98; Danger of the Dead Tooth, 166; Consequences of Disease, 195; Caries, 230; Dentistry Bill, 398; Dentistry as a Profession, 571
Derby Sanatorium, 508, 595
Detroit, Nurse Training for, 418
Devon Joint Committee, 469
Devon Nursing Association, 233
Diathermy, 501
Dick, Sir James N., 425
Dietary of Hospital Patients, Supp. Jan. 12, July 24
Discharged Disabled Soldiers (*see also* Ministry of Pensions), 310, 475, 490, 516, 610
Disease and the Passing Years, 245
Dispensing (*see also* Drugs): Industrial Training for Pharmacists, 185; Registration without Examination, 427
District Nurses, Outer Hebrides, 50
Divorce Controversy, The, 122
Doctor and His Patient, The, 274
Doctor's Heroism, 251
Domestic Animals and Human Infection, 540
Domestic Service in Hospitals, Supp. Aug. 7, Aug. 28, Sept. 11, 537, 629
Drake, Mr. C. W., 53
Drugs (*see also* Dispensing): The Week's Market in, 6, 32, 56, 82, 108, 134, 164, 192, 218, 244, 268, 294, 317, 354, 378, 404, 428, 451, 478, 500, 522, 544, 573, 596, 618, 636
Drunkenness Statistics, 487
Dublin: Financial Position of Hospitals, 265
Duchess of Bedford's Hospital, 281
Dudley, Countess of, 364
Durham County Council, 164, 610; Nursing Association, 394
Dysentery, New Factor in Treatment, 597
Dyspeptics, Cooking for, 628

Ear, Diseases of the Internal, 502
East Sussex County Nursing Federation, 418
Eccles, Mr. McAdam, 132, 268
Edinburgh, School of Cookery, 416; Medical Missionary Society, 534
**EDITOR'S LETTER-BOX, THE:**
Baby Clinic Experiences, 362
Brussels Health Congress, 125
Burdett, Sir Henry, The Late, 181
C.M.B. Candidates, 259
Contributions from Patients, 21
De La Rue Scheme of Medical Service, The, 441, 467
Dispensaries for Hop-pickers, 629
Domestic Service in Institutions, 557, 629
Employee's Industrial Views, An, 125
Experiments on Animals, 283
Gift of Expression, The, 649
Gravesend Hospital, 493
Guest Hospital, Dudley, 235
Hospital Domestic Service, The Status of, 514, 535
Hospital Saturday Fund, The, 181, 259
Hospital Sunday, The Story of, 396

EDITOR'S LETTER-BOX, THE—*continued.*
Hospital Survey, Lessons of the, 419
Income-Raising Device in the Antipodes, 468
Industrial Hygiene, 181
Lay Contributor, The, 259
Making the Public Understand, 557, 576, 611
Male Nursing, 468
Medicine's Opportunity, 99
Memorial Hospital, Mildmay Park, 493
Mental Patients in War Hospitals, 649
National Health and Modern Problems, 419, 442, 467, 493
"Needs Must When—" 576, 611
Norfolk and Norwich Hospital, 650
Nurse-Training in Special Hospitals, 209
Nurses and an Eight-hour Day, 283
Nurses' Hours, 283
Nursing Certificate, The Value of a, 513
Patients' Payments, 309
Poor-Law Nurses and Trade Unions, 535
Private Nurse's Earnings, A, 181, 235
Professional Dualities, 209
Professional Secrecy and Professional Confidences, 395
Safe Milk, 21
St. George's Hospital, Position of, 441
Sanitary Blocks of Hospitals, 21
Scrapping the Trained Nurse, 22, 47
South Africa, School Medical and Nursing Services in, 576
State Assistance to Voluntary Hospitals, 73
Status of Hospital Domestic Service, 557
Status of the Trained Nurse, The, 259
Telephone Box, The Dangerous, 420
Tied Houses in Surgical Practice, 182, 209
Union in the Nursing Profession, 396
University College Hospital and the Royal Ear Hospital, 125
Venereal Prevention, 443, 468, 513
Edmonton Board of Guardians, 106, 537, 544
Educated Women as Nurses, 440
Egypt, Nurses in, 398
Eighteenth-Century Methods, 254
Employers and the Ex-Service Man, 518, 555
Employers' Subscriptions and Smaller Hospitals, 497
Encephalitis Lethargica, Acute, 8
Epidemics, Fight Against, 178
Epsom College, 401
Essex County Nursing Association, 41
Eugenics, A Question of, 301; Mr. Hilaire Belloc and, 485
Eurhythmics and Health, 603
Ex-Service Men, The Appeal for, 518, 552

Factories, Welfare in, 323
Factor in Disease Control, A, 28
Father's Responsibilities, The, 488
Fatigue, Blood Pressure and, 429
Federation of Medical and Allied Societies, 131, 425, 475, 481
Fever Nurses' Association, 257
FOOD (*see also* Milk)
Food Education Society, 175; Food Values and Common-sense, 298, 452; Surprising Category of Dangerous Foods, 411; A Rare Case of Poisoning, 524; Cookery and Food Exhibition, 647.
Forthcoming Events, 102, 212, 385
Forty-eight Hour Week, A, 172, 230, 353
From Across the Seas, 287
Full-time Teacher, A, 188
Future of Medicine and the Medical Profession, The, 561

Gallio of Commerce, The, 4
Gastric Function, The, 632
Gastric Ulcers, 165
Gelligaer Isolation Hospital, 417
General Council of Medical Education, 275
General Medical Council, 349, 370
General Nursing Council, 118, 127, 153, 234, 415, 510, 646
General Nursing Council for Scotland, 100, 330, 332, 532
General Practice, 563
General Practitioners and the New Health Bill, The, 356
General Practitioner's Handicap, The, 351
George, Mrs. Lloyd, 541
Gifford House, Roehampton, 40
Gift of Expression, The, 630, 649
**GIFTS AND BEQUESTS**, 22, 47, April 17, vi; 90, 116, May 15, iv, 211, 288, 397, 515, 535, Sept. 4, xx, 612
Glasgow, Hospital Sunday, 30; Police Force, 521
Glass Oxygen Rooms, 498
Gloucestershire Medical Service Scheme, 105
Gold in His Boots, 216
Gonorrhœa (*see* Venereal)
Goodall, Dr. J. Strickland, 131
Gorgas, Surg.-General, W. G., 377, 407
Government Forms, 190
Graded Medical Service, 352
Grangethorpe M. of P. Hospital, 13
Grate, The Domestic, 413
Greenwich Borough Council, 108
Gregoe-Colmore, Mr. W. B., 353
Gunshot Wounds, 23, 49, 74
Guyon, Professor, 447
Guy's Hospital Nurses' League, 281

Haig, Lord, 518, 552, 555
Half-pay Officers, Medical Treatment of, 615
Hamer, Dr. W. H., 545
Hammersmith, Borough Council, 108
Hampstead Guardians, 393
Haverfield, The Hon. E., 15
Hawthorne, Dr. C. O., 143
Health and Artificial Lighting, 57
Health Versus Disease, 279
Health Visitors, 233, 437, 494, 509, 645
Heard in the Corridor, 611
Hearson, Mr. C., 54
Heart, Surgery of the, 500
Herefordshire County Nursing Association, 648
Herring, Mr. George, 321
Hertfordshire County Nursing Association, 209
Higgins, Dr. W. J., 251
Highbury and Uffculme Hospital for ex-Service men, 4
Hillier, Nurse, 308
Hocking, Mr. F. A., 288
Holiday Tax, The, 435
Home Life, 180
Honorary Medical Staffs, Payment of, 259
**HONOURS:**
Birthday Honours, 265
Hospitals and the Honours List, 51, 79
Order of the British Empire, 66, 79, 173, 463
Royal Red Cross, 14, 39, 118, 205, 255, 305, 363, 392, 463, 509
Royal Red Cross, Second Class, 608
Horsley, Sir Victor, A Memorial to, 315
**HOSPITAL AND INSTITUTIONAL NEWS**, 3, 29, 53, 79, 103, 131, 161, 189, 215, 241, 265, 291, 315, 351, 375, 401, 425, 447, 475, 497, 519, 541, 573, 593, 613, 633
Hospital and Institutional Results, 99
**HOSPITAL ARCHITECTURE AND CONSTRUCTION:** The New Era, 37; Royal Devon and Exeter Hospital, 621
Hospital Crisis, The (*see* Crisis)
Hospital from Inside, A, 11
Hospitals and Missions, 427
Hospitals and the Honours' List, 51, 79

**HOSPITALS, INSTITUTIONS, ETC.:** (Poor law, War, and Temporary Hospitals, *see under* individual names)
METROPOLITAN:
Alexandra Hospital for Children, 191, 218, 476
American Hospital, 163, 316
Belgrave Hospital for Cildren, 175, 243
Bethlem Hospital, 6, 132
Bolingbroke Hospital, 191, 634
British Home and Hospital for Incurables, 317
British Hospital for Women and Babies, 76, 186
Cancer Hospital, 48, 163
Charing Cross Hospital, 210
Chelsea Hospital for Women, 260, 439
City of London Hospital for Diseases of the Chest, 100
City of London Maternity Hospital, 256
East London Hospital for Children, 235
Elizabeth Garrett Anderson Hospital, 119, 392
Evelina Hospital, 243
Florence Nightingale Hospital, 234
Foundling Hospital, 81
Freemasons' Hospital, 3
Great Northern Central Hospital, 100, 119, 206, 288, 396, 401, 407, 418, 448, 544, 616
Guy's Hospital, 281, 421, 445, 465, 511, 615
Hampstead General Hospital, 211
Hospital for Consumption, 491, 494
Hospital for Diseases of the Throat, 573, 635
Hospital for Sick Children, 260, 308
Italian Hospital, 325, 616
Jewish Hospital, The, 107
Kensington and Fulham General Hospital, 378
King's College Hospital, 469, 477, 536
London Fever Hospital, 235, 377
London Homœopathic Hospital, 25, 32
London Hospital, The, 190, 240, 254, 257, 260, 265, 353, 393, 591, 599, 607, 615
London Lock Hospital, 4
London School of Tropical Medicine (*see* Seamen's Hospital)
London Temperance Hospital, 25
Manor House Orthopædic Hospital, 207
Metropolitan Hospital, 48, 191, 192, 311
Middlesex Hospital, 53, 218, 408
Miller Hospital, 377, 635
Mount Vernon Hospital, 25
National Hospital for Diseases of the Heart, 76
National Hospital for the Paralysed and Epileptic, April 10, vi, 207, 265, 293, 310, 377, 500
Newport, Royal Gwent Hospital, 191
Norwood Cottage Hospital, 208
Paddington Green Hospital, 273
Poplar Hospital, 48, 55
Prince of Wales's Hospital, 626
Queen Charlotte's Hospital, 76, 242
Queen's Hospital for Children, 189, 206, 260
Royal Dental Hospital, 3, 48, 617
Royal Free Hospital, 478
Royal Hospital for Diseases of the Chest, 76
Royal Hospital for Incurables, Putney, April 17, vi, 594, 635
Royal Maternity Charity, 76
Royal National Orthopædic Hospital, 48, 402
Royal Surgical Aid Society, 522
Royal Waterloo Hospital, 100, 306
Royal Westminster Ophthalmic Hospital, 48
St. Bartholomew's Hospital, 92, 120, 242, 280, 282, 353, 421, 466, 647
St. George's Hospital, 68, 76, 267, 478

Hospitals, Etc.—*continued.*
St. Luke's Hospital, 428
St. Mary's Hospital, 242, 421, 427, 444, 633
St. Mary's Hospital, Plaistow, 65
St. Paul's Hospital, 267
St. Thomas's Hospital, 199, 258, 260, 322, 421, 538, 553, 634
Samaritan Free Hospital, 364
Seamen's Hospital Society, 242, 595
South London Hospital for Women, 260, 465, 647
University Hospital, 48, 80, 206, 296, 307
Victoria Hospital for Children, 260
Weir Hospital, Balham, 378
West London Hospital, 54, 235, 418
Westminster Hospital, 16, 265
Provincial, Scottish, and Irish:
Aberdare General Hospital, 80
Aberdeen: Royal Infirmary, 354; Royal Hospital for Sick Children, 133, 354
Aldershot Hospital, 267
Alton Cripples' Hospital, 55, 317
Aylesbury, Royal Bucks Hospital, 634
Bangor, Carnarvonshire Infirmary, 192
Barnsley, Beckett Hospital, 308
Barnstaple, North Devon Infirmary, 520
Barrow, North Lonsdale Hospital, 234
Bath, Royal United Hospital, 403
Batley and District Hospital, 393
Beddington, Royal Female Asylum, 107
Bedford County Hospital, 498
Belfast, Royal Victoria Hospital, 56, 403
Bideford General Hospital, 4
Birmingham: Eye Hospital, 6; Hospital for Women, 30, 76; Ear and Throat Hospital, 41; General Hospital, 68, 76, 444, 476, 633; Queen's Hospital, 542, 634
Blackburn Infirmary, 67
Blaina Hospital, 636
Bournemouth, Royal Victoria Hospital, 244, 521
Bradford, Royal Infirmary, 107, 244
Bridgend Cottage Hospital, 32
Brighton: Royal Sussex County Hospital, 5, 192, 378
Bristol: Voluntary Lock Hospital, 4; Children's Hospital, 191; General Hospital, 403, 596; Royal Infirmary, 427
Bury St. Edmunds, West Suffolk Hospital, 76, 615
Caerphilly, Miners' Hospital, 634
Cambridge, Addenbrooke's Hospital, 542, 635
Canterbury, Kent and Canterbury Hospital, 478, 520
Cardiff, King Edward VII. Hospital, 5, 133, 215, 217, 266, 401, 475, 471, 522
Carnarvonshire and Anglesey Infirmary, 402
Chailey, Heritage Craft Schools, 94
Chelmsford, Chelmsford and Essex Hospital, 55
Cheltenham General Hospital, 76
Chester Royal Infirmary, 22
Chesterfield, North Derbyshire Hospital, 5, 499
Chichester, Royal West Sussex Hospital, 218
Coventry and Warwickshire Hospital, 267, 336, 477, 618
Cowes, Frank James Memorial Cottage Hospital, 489
Crickhowell, Ivy Tower War Memorial Hospital, 402
Cumberland Infirmary, 627
Cwmparc Cottage Hospital, 293
Derby, Royal Infirmary, 76, 500
Dollis Hill Hospital, 30
Doncaster General Infirmary, 105
Dublin: Rotunda Hospital, 189; National Children's Hospital, 266
Dudley, Guest Hospital, 191, 477, 610
Earlswood, Royal Earlswood Institution, 76
Enfield Cottage Hospital, 476
Evesham Cottage Hospital, 31

Hospitals, Etc.—*continued.*
Edinburgh Royal Infirmary, 391, 402, 428, 609
Exeter, Royal Devon and Exeter Hospital, 282, 511, 520, 621
Glasgow: Western Infirmary, 79; Hospital for Sick Children, 394
Gloucester Royal Infirmary, 55, 491
Gravesend Hospital, 76, 449
Great Yarmouth Hospital, 76
Harrogate: Babies' Hospital, 31; Infirmary, 108
Hastings, East Sussex Hospital, 243
Hendon, Colindale Hospital, 416
Hull General Infirmary, 16
Ipswich, Essex County Hospital, 76, 498
Keighley Victoria Hospital, 520
Lancaster Infirmary, 466
Leamington, Warneford Hospital, 76, 133
Leeds, Royal Infirmary, 3, 105
Leicester, Royal Infirmary, 88, 126, 244, 287, 462, Sept. 18, vi
Lincoln County Hospital, 82
Liverpool: Dental Hospital, 31; Royal Southern Hospital, 76, 94, 158; Maternity Hospital, 76; Royal Infirmary, 92; David Lewis Hospital, 404, 444; Stanley Hospital, 521
Llanelly Hospital, 573
Lowestoft and North Suffolk Hospital, 394
Maesteg and District Hospital, 108
Maidstone, West Kent Hospital, 93, 134, 593
Manchester: Ancoats Hospital, 16, 56; Children's Hospital, 56; Salford Hospital, 403
Margate: Royal Sea Bathing Hospital, 476; Princess Mary Hospital, 628
Marlborough, Savernake Hospital, 29
Merthyr Hospital, 544
Middlesbrough, North Ormesby Hospital, 308
Mildenhall Cottage Hospital, 294
Monkwearmouth and Southwick Hospital, 520
Monmouth, Convalescent Home, Sept. 25, vi
Neston Cottage Hospital, 450
Newark Hospital, 366, 393, 438
Newcastle-on-Tyne: Orthopædic Hospital, 82; Royal Victoria Infirmary, 428, 475, 512, 541, 616
Newport, Royal Gwent Hospital, 133, 520
Northampton, General Hospital, 618
Norwich, Norfolk and Norwich Hospital, 76, 163
Nottingham, General Hospital, 54, 477, 544
Otley Infirmary, 617
Oxford, Radcliffe Infirmary, 500
Plaistow, Maternity Charity, 438
Plymouth, Devon and Cornwall Ear and Throat Hospital, 267
Pontypool Hospital, 288
Portsmouth General Hospital, 618
Preston Royal Infirmary, 42
Reading, Royal Berkshire Hospital, 5, 268
Rhondda Hospital, 404
Richmond, Royal Hospital, 76
Rochester, St. Bartholomew's Hospital, 595
Romford, Victoria Cottage Hospital, 5
Ryedale, Cottage Hospital, 636
Salford Royal Hospital, 597
Salisbury Infirmary, 80, 629
Saltash, St. Barnabas' Hospital, 191
Sheffield, Royal Infirmary, 422
South-Eastern Hospital for Children, 515
Southport Infirmary, 354
Stockport Infirmary, 450
Stockton Hospital, 595
Stoke-on-Trent, North Staffordshire Infirmary, 635
Stratford-on-Avon Hospital, 81, 366
Surbiton Hospital, 80
Sunderland, Royal Infirmary, 404
Swansea Hospital, 3, 208, 498
Truro, Royal Cornwall Infirmary, 595
Walmer and Deal Hospital, 608

Hospitals, Etc.—*continued.*
Ventnor, Royal Hospital for Consumption, 134, 210
Walsall General Hospital, 34, 76
West Bromwich Hospital, 316, 635
Weymouth, Dorset Eye Infirmary, 499
Whitehaven, West Cumberland Infirmary, 208, 477
Whitstable Cottage Hospital, 294
Wigan, The Royal Albert Edward Infirmary, 15, April 17, vi
Winchester, Royal Hants County Hospital, 80, 399, 403, 410, 490, 533
Windsor, King Edward VII. Hospital, 80, 82
Woodhall Spa, Alexandra Hospital, 521, 618
Yarmouth Hospital, 596
York: County Hospital, 292; Maternity Hospital, 282
Colonial, U.S.A., etc.
Auckland, Public Hospital, 207
Bombay, Catholic General Hospital, 449; Children's Hospital, 471
Bloemfontein, National Hospital, 558
Cape Town, Denis Buxton Memorial Hospital, 286; Somerset Hospital, 286
Davos, Queen Alexandra Sanatorium, 15, 29
Johannesburg: Children's Hospital, 548; General Hospital, 622
Marseilles, British Hospital, 163
Massachusetts General Hospital, 634
New York, Presbyterian Hospital, 596
New Zealand: Taranaki Hospital, 287; Wellington General Hospital, 536
Paris, British Hospital, 294
Pittsburgh, Pennsylvania Hospital, 82
Poona, Infectious Diseases Hospital, 428
Sydney: Woman's Hospital, 281; Royal Prince Alfred Hospital, 635
Toowoomba Hospital, 596
Hospital Fête Story, A, 559
Hospital Finance, 285, 304
Hospital Magazines and Journals, The, 421
Hospital Saturday Fund, 189, 197, 225, 316
Hospital Sunday Fund, 313; Special Number, 337, 351, 408, 444; Distribution, 473, 492, 495, 508, 510
Hours of Employment Bill, 256, 262
Housing, 156, 157, 168; World Conference, 275; Scandal of Empty Houses, 604; Domestic Arts and, 643
Howe, Miss A. E., 42
Howell, Mrs., Pension for, 163
Hull, Asylums Committee, 164
Hungary: Infant Mortality, 499
Hygiene and the Elementary School Teacher, 284; The Study of, 542

Illegitimacy: In Monmouth, 29; Maternity Homes, 164; The Bill, 177; Hostels for Mothers, 277; The Ugly Word, 389
Imperial Cancer Research Fund, 447
Imperial War Museum, 292
Imperial War Relief Fund, 634
Income-Raising Device in Australia, 448, 458
Income Tax (*see also* Tax-Free Benefactions), 115, May 15, iv; Hospital to Pay, 189; Charitable Contributions, 212, 471
Incorporated Association of Hospital Officers, 161
Incorporated Society of Trained Masseuses, The, 312, 331, 365, 511
Increased Spirit Duties, 402
India: Hospital Problems in, 380, 454; Russian Red Cross in, Sept. 4, xx
Indian Degrees, 102
Indian Medical Service, 212, 471
Industrial Court, Hospitals and the, 409
Industrial Disease, 241
Industrial Fatigue, 644

Industrial Medicine, 270, 633
Industrial Welfare, 121
**INFANT WELFARE AND MATERNITY:** (*see also* Midwives and Public Well-Being, The): Economics and Motherhood, 17, 20; Isle of Dogs, 42; Education Act, 46; Employment, 46, 72; India, 72, 81, 93, 217; Homes for Mothers, 97; Baby Week, 123, 277, 391; Crippled Children 155, 157; Children's Rights, 228; National Congress, 252; York, 276; Liverpool, 277; Chelmsford, 294; Experiences, 303; Manchester, 324; Birmingham, 388; Walthamstow, 422; Bombay, 449; Home of Rest, Sydenham, 491; Welsh Mothers, 606; Workhouses, 643
Infectious Disease in Children, 63; Reports, 620
Infirmary Wards, Unrest in, 422
Influenza, 196, 232, 258, 266, 400
Inglis, Elsie, Memorial, 119
Inman, Mr. Philip A., 189
Insane (*see* Mental)
**INSTITUTIONAL FACTS AND FIGURES** (*see* The Institutional Worker in the Supplement to "The Hospital")
Institutional Library, The (*see* Book World)
Institutional Needs, 238, June 12, vi, 311
Insurance, Accident and Illness, for Nurses, 331
International Health Council, 92, 97
Invalid Children's Aid Association, 67, 530
Investigation Home, An, 105
Isle of Wight County Council, 627

Keen, Mrs. 648
Keith, Professor A., 421
Kenilworth Hospital Saturday, 573
Keyser, Mr. L., 399
Kidnapping of a Nurse, 648
King Edward's Coronation Fund for Nurses, 234
King Edward's Hospital Fund for London, 47, 130, 137, 313, 315, 328, 376, 397, 448, 455, 495
KING, THE, 13, 19, 40, 291, 391, 416, 425, 427
King's College for Women, 465, 490, 531
Kingston Infirmary, 417, 440, 533
Knutsford, Viscount, 132, 254

Lambeth: Board of Guardians, 14, 163, 216, 258; Infirmary, 354, 514; Borough Council, 366, 512
Lambeth Medical Degree, 377
Laryngitis, The Severer Forms of, 171
Lawlor, Mr. R. T., 185
Lay, Major Tudor, 134
League of Mercy, The, 416
League of Remembrance, The, Supp. Sept. 18
Legal Notes for Institutional Workers, 73, 432
Leicester, Saturday Hospital Society, 12, 521
Leigh, Lady, 66
Leprosy, A Cure for, 426
Lewes Workhouse Infirmary, 68
Lewis, Dr. T., 598
Lewisham Guardians, 556
Limited Companies and Subscriptions, 267
Lincoln Union Infirmary, 532, 565
Lister Institute, 215
Lister Memorial Fund, 448
Literature and Public Welfare, 95
Little Hospitals, The Dilemma of the, 489
Liverpool: University Appeal, 78; Council of Voluntary Aid, 107, 595, 642; Practical Co-ordination, 268; New Convalescent Home, 449
Llandilo Guardians, 519
Lloyd, Miss K. G., 465
Local Government Board (*see* Ministry of Health)
Logie, Dr. J. S. S., 215, 225
London, The Health of, 545
London County Westminster and Parr's Bank, Ltd., 414
London County Council, 63, 119, 131, 394, 417, 545, 626
London Health Services, 220
London, Healthy, 436
London Juvenile Courts Bill, 324
London School of Medicine for Women, 234, 354
London Teachers' Association, 32, 417
London University, 215
Lord Dawson's Scheme, 239
Lord, Sir Riley, 541
Lotteries, 54
Louth Flood, The, 251
Luard, Miss K. E., 465, 647
Lucas, Mr. Hedley, 542, 634
Lyle, Mr. C. E. Leo, 594
Lyster, Lord, 80

MacAlister, Sir Donald, 275
McCarthy, Dame Maud, 365
Macclesfield Infirmary, 440
McMaugh, Sister H. H., 68
Maddox, Dr. E. E., 390
Madness (*see* Mental)
Makins, Sir George, 142
Malaria, 131; Survey in England, 355, 461
Malta, Hospital work in, July 10, vi
Man and the Higher Fungi, 135
Manchester: The King's Visit, 19; Milk Supply, 98; Public Health Department, 123; Saturday Fund, 191; Crumpsall Infirmary, 609
Maple, Sir Blundell, The Late, 80
Marquardt, Miss F. E., 258
Marylebone: Borough Council, 6; Infirmary, 365
Massage Establishments, 179
Maternity (*see also* Infant Welfare and Midwives); Birmingham Scheme, 30; More Hospitals Needed, 228
Mathews, Miss Geraldine, 440
Matron's Administrative Staff, Duties of, Supp. June 19, July 24
**MATRONS' AND SISTERS' DEPARTMENT**, 13, 39, 65, 91, 117, 153, 173, 205, 231, 255, 279, 305, 329, 363, 391, 415, 437, 463, 489, 509, 531, 553, 575, 607, 625, 645
Matthews, Major L. W., 30
Maudsley Hospital, 519
Mayflower Celebrations, 608
Mayo, Dr. C. H., 376
Measles, 546
Medical Curriculum, The, 579
Medical Defence Union, 616
Medical Domesday Book, 87
Medical Fashions, 350
Medical Honoraria, 241
Medical Research Committee, 3, 69
Medical Schools, Crowded, 160
Medical Student, What Happens to the, 190; His Work, 581
Melbourne Hospital Saturday and Sunday Funds, 6
Melton Mowbray: Suggested Cottage Hospital, 497
Mental: Uncertifiable Discharged Men, 26; Studies in Inefficiency, 58; Supervision of Defectives, 164; Insanity and Justice, 219; Hospital Charges, 449; Administration of Lunacy Acts, 455; Mental Hospitals Association, 447; Mental Hospital Service, 565; Incipient Disease, 613
Merthyr Union Infirmary, 439, 465, 555
Mesopotamia: Health of Troops, 482; Nursing In, 627
Metropolitan Asylums Board, 427, 475, 532, 533, 594, 620, 626
Metropolitan Nursing Association, Supp. May 22
Michelli, Mr. P. J., 144
Middle Age, The Cult of, 434
Middle Class, Hardships of the, 387
Middlesex County Council, 317
Midwives: Statistics, 152; Scholarships, 175; General Medical Council, 264; Doctors' Fees, 317; Salaries or Fees? 449; Scholarships in Kent, 491; Scottish Roll, 534; Salaries at Bath, 610
Milk: Cinema Film, 71, 180; Sterilisation, 161, 247; Grading, 226; Certified, 292; Milk and Dairies Bill, 507; A New Combine, 530
Millbank Hospital, 291
Miller, Canon, 319
Mine Rescuers, The Strike of, 112
Miners' Nystagmus, 26
Minister of Labour: Joint Industrial Council, 107; Hours of Employment Bill, 352
Minister of Pensions, 40, 310; Nursing Service, 365, 439
Ministry of Health: Non-Resident Nurses, 14; New Bill, 356, 517; New Secretary, 79; Medical Remedies, 136; Staff of Outdoor Medical Officers, 167; The "New Hospitals," 214, 356; Consultative Council on Medical and Allied Services, 221, 233, 239, 241, 253, 256, 257, 265, 273; for Wales, 242; Voluntary Hospitals, 77, 211, 288, 315, 370, 393, 426, 440; General Nursing Council, 415; Outdoor Medical Staff, 420; Health Services Vote, 424, 435; Miscellaneous Provisions Bill, 517, 550, 594; List of Homes and Hospitals, Aug. 28, vi; Annual Report, 615; Infectious Diseases, 620; Call for Nurses, 625
Missions, Hospitals and, 427
Modern Universities, The, 89
Money-Lenders and Public Bodies, 268
Mons, Ceremony at, 208
Morley, Mr. Howard, 131
Morphia, 288
Morris, Mr. E. W., 81, 376
Morris, Sir Malcolm, 475, 481
Mort, Colonel, 106
Mosquitoes, 542
Motherhood (*see* Infant Welfare)
Mothers' Defence Association, 101
Motor-Car Taxation, 161
Motors for Medical Officers, 215
Mystery, An Unsolved, 400

Nation's Fund for Nurses, The, 329, 437
National Association for the Prevention of Infant Mortality, 277, 626
National Association for the Prevention of Tuberculosis, 573
National Council of Social Service, 71
National Farmers' Union, 317
National Food Journal, 353
National Health Assurance: Higher Sick Benefit, 266; New Benefits, 387; Cost of, 444; Referee Consultants, 482
National Health Week, 66, 69
National Institute of Psychology and Physiology, 53
National Physical Laboratory, The, 499
National Relief Fund, 272, 316, 541
National Responsibility, A, 517
National Society of Operative Printers, 243
Nationalisation, 190
Nerves, The Surgery of, 542
Nervous Disease, Early Symptoms and Signs of, 547; New Journal, 560; New Clinic, 615
Neurasthenics: Colonies for Incurables, 525
New Buildings Contemplated, 516
Newcastle: Cathedral Nursing Association, 533
New End Hospital, Hampstead, 608

"New Hospitals," The, 214
Newington House, 466
New Poor, Defining the, 11; Hospital and Nursing Home Accommodation, 177
New South Wales: Nursing Conditions, 466
New York Nurses, 556
Newsvendors' Benevolent and Provident Institution, Sept. 4, xx
New Zealand: Hospital Board, 472; Nursing Conditions, 536
Nigeria, Medical Officers in, 230
Nightingale, Florence, Centenary, 161, 170, 174; Award of Medals, 256; Cinema Film, 609
Night Work, Women and, 301
North London Nursing Association, 120
North Staffordshire Cripples' Aid Society, Supp. May 22
Norwich: Poor-Law Infirmary, 131; District Nursing Association, 331
Notifiable Diseases, 131
Nottingham: Ministry of Health and the Corporation, 276
Nurse-Apprentice, The, 531
Nursery Schools, The Purpose of the, 229
Nurses' Insurance Society, 282
Nurses, Scarcity, 118, 231
Nursing Administration, The Uses of Freedom in, 415
Nursing and Midwifery Conference, 306, Supp. July 3, 10
Nursing Association, Winding Up of a, 398
Nursing Homes: For the New Poor, 79; The "Scandal," 532

**OBITUARY:** The Hon. E. Haverfield, 15; Mrs. Humphry Ward, 17; Miss Alice Bradford, 26; Dr. H. Treub, 53; Mr. C. Hearson, 54; Adeline Duchess of Bedford, 92; Sir Henry Burdett, 129; Miss Bessie Carley, 175; Miss Sophia Palmer, 233; Dr. R. S. Savage, 286; Countess of Dudley, 364; Surgeon-General Gorgas, 376, 407; Sir A. Dempsey, 447; Professor Guyon, 447; Mrs. Batten, 491; Professor Politzer, 522; Sir Riley Lord, 541
Obstetric Training of the Medical Student, 264
O'Donoghue, Rev. E. G., 6
On the Track of a Ward Maid, 24
Operating Theatre, Ideals in the, 560
Ophthalmic Cases, An Analysis of, 560
Opium, 288
Optical Appliances, 102
Ormsby, Sir Lambert, 266
Orthopædic Appliances, 211
Osler, Sir William, 53, 113, 497
Overseas Nursing Association, 273, 365, 370
Oxford: Women Students, 418
Oxygen, 635

Paddington Guardians, 308
Paget, Lady Muriel, 120
Palmer, Miss Sophia, 233
Pancreas, Diseases of the, 33
Panter, Mr. G. G., 423, 431
Parenthood, Economics and Knowledge, 124
Parents' Advisory Bureau, 106
Paris: Surgical Congress, 401
**PARLIAMENT AND THE PROFESSIONS,** 26, April 10, vi, 102, 128, 152, May 15, iv, 212, 230, 288, 310, 325, 370, 398, 422, 444, 471, 482, 516, 538, Aug. 28, vi
Parliamentary Vote for Women, 41
**PASSING EVENTS AND TOPICS,** April 10, vi, April 17, vi, 100, 152, 288, 311, July 10, vi, 422, 471, Sept. 4, xx
Patent Medicines, 136
Patient's Point of View, From the, 559
Pay Hospitals, Nursing Conditions in, 13
Payments by Patients, 211, 448, 476, 558, 591, 615
Peace Day Anniversary, 439
Penal Reform League, 510
Pencils as Carriers, 54
Pensions for Naval and Military Medical Men, 128
People's League of Health, 157
Pestilential Streams, 507
Peterkin, Miss, 91
Pharmaceutical Council of Great Britain, 560
Pharmaceutical Society, The, 427
Physiology, The Teaching of, 598
Plaster Work in General Practice, 271, 379
Plymouth: Puerperal Fever in, 330, 500; Infirmary, 393
Politzer, Professor, The Late, 522
Pontyclun: Ambulance Contest, 628
Poor-Law Association, The, 404
Poor-Law: A Debatable Plan, 45; Children, 186; Almoners, 444; Nurses' Rations, 512; Patients' Accommodation, 516; Statistics, 521; Training of Nurses, 554; Medical Officers' Association, 560; Medical Service, 570; Nurses' Societies, 646
Population and Parenthood, 202
Port Sanitary Authority, 637
Portsmouth: Municipal Maternity Hospital, 176
Post-Graduate: Assistance, 1; Teaching in London, 568
Poultry-Keeping in Hospitals, 124
Pound Day, A Successful, Supp. June 5
Prejudice, The Passing of, 507
Pretyman, Mr. E. G., 498
Prevention After Cure, 213
Preventive Medicine, 111
Prince Albert, 189, 206
Prince of Wales, The, 3
Princess Alice, 391
Princess Arthur of Connaught, 364, 417
Princess Beatrice, 490
Princess Helena Victoria, 232
Princess Mary, 174
Prisoners of War Library, A, 503
Prisons: Medical Officers, 398; Nursing Staffs, 482; Reform, 505
Private Hospitals in Scotland, 216
Probationers: Changes, 173; An Entrance Examination, 463; who Abandon Training, 645; in European Countries, 647
Professional Classes, A Hospital Trust for, 519
Professional Dualities, 134
Professional Union of Trained Nurses, 127
Progress, The Idea of, 251
Proprietary Medicines Bill, 474, 486
Public Health: New Health Provisions, 20; In Ireland, 362; Medical Service, 567
Public Pharmacists' Association, 100, 152
**PUBLIC WELL-BEING, THE:** 17, 43, 69, 95, 121, 155, 177, 201, 226, 251, 274, 301, 323, 359, 387, 411, 433, 459, 485, 505, 528, 550, 574, 603, 623, 641
Publicans and Delirium Tremens, 5
Pussyfoot in French India, 32

Q.A.M.N.S.I., 232
Quack Medicines, 316
Queen, The, 40, 391, 416, 511
Queen Alexandra, 91, 187, 232, 364
Queen Elizabeth, 208
Queen Mary's Hostel, 440
Queen Mary's Needlework Guild, 206
Queen Victoria's Jubilee Institute for Nurses, 15, 392, 464

Railway Fares, 646
Railway First Aid, 94
Rathbone, Mrs. H., 449
Rats, How to Make a Building Rat-Proof, 243
Raw, Dr., 402
Reading Historical Pageant, 232
Recent Official Publications, 262, Sept. 18, vi
Recreation at School Age, 528
"Red" Army, 40
RED CROSS (*see also* V.A.D.): Central Europe, 118; To Help Voluntary Hospitals, 132, 187, 199, 232, 236; Canadian, 176; Surrey, 176; In the Shires, 233; Hospital Survey, 199, 236, 291, 447; Ambulance and Health Services, 241; Library, 317; The Appeal, 447; King Edward's Fund, Distribution of Surplus Funds, 448, 455, 464; In India, 471; Australian, 533; Peace Report, 541; Our Day, 554; Tour of Sir A. Stanley and Sir N. Burnett, 593, 616, 617, 631, 639, 652
Reform of Medical Teaching, The, 349
Registrar-General, The, 400
Rent Bill, 258
Research and the Medical Student, 569
Restaurant, Perils of the, 605
Research Defence Society, 53, 315
Retford, Soldiers' Home, 416
Riddell, Miss M. S., 415
Robbing the Hospitals, 353
Roberts, Mr. G. Q., 145, 261
Robinson, Sir Wm., 79
Rockefeller Foundation, 297, 307
Romford Infirmary, 257
Ross, Sir Ronald, 131
**ROUND THE HOSPITALS,** 13, 39, 65, 91, 117, 153, 173, 205, 231, 255, 279, 305, 329, 363, 391, 415, 437, 483, 489, 509, 531, 553, 607, 626, 646
Royal Academy, The, 182
Royal Air Force, Nursing Service, 490
Royal Army Medical Corps, 302, 565, 59[illegible]
Royal British Nurses' Association, 336
Royal College of Surgeons, 54
Royal Institute of Public Health, 234
Royal Medical Benevolent Fund, 241
Royal National Pension Fund for Nurses, 306, 364, 369, 391
Royal Naval Medical Service, 564
Royal Sanitary Institute, 206, 414, 433, 459, 464, 556
Royal Society, The, 224
Royal Victorian Trained Nurses' Association, 93
Rubber Growers' Association, 265
Russia: Relief Fund for Women and Children, 331; Red Cross, 331

St. Andrew's Ambulance Association, 601
St. Anthony's Pigs, 80
St. Dunstan's Hostel for Blinded Soldiers, 16. 54, 93, 133, 217
St. John Ambulance Association, West Riding Branch, 596
St. Kilda, 196
St. Pancras: Guardians, 331; Infirmary, 354
Salad Dressing, 512
Salaries, Sisters' and Nurses': Preston Royal Infirmary, 42; Blackburn Infirmary, 67
Sanitary Science and Preventive Medicine, 548
Sanitation, Public, 389
Savage, Dr. R. S., 286
Saving the Children, 552
School Children to Help Hospitals, 132
School Play Centre, 644
Scoliosis, Crawling Exercises for, 536
Scotland, Finance of Voluntary Hospitals, 475; Motor Ambulance Service, 601
Scrapping the Trained Nurse, 12
Sea, Operations in War Time, 310
Second-Hand Book Fund, A, 477
Seely, General, 134
Self-Determination for Nurses, 278
Sex Hygiene for the Young, 485

Sharpe, Miss S., 416
Sheffield, Historical Pageant, 232
Sheffield: Lodgmoor Hospital, 258; Shortage of Nurses, 282
Sheffield Street Hospital for Women, 230
Shell-Shock, 693
Sheriff-MacGregor, Miss, 118
Should a Doctor Tell? 351, 359
Siberia, American Red Cross, 40
Sites of London Hospitals, 325, 360
Smallpox, 53, 194, 248
Smoke Pall, The, 226; Cost of, 605
Smoking in Hospital Wards, 64
Sneer at Doctors, A, 252
Society for the Visitation of the Sick, 268
Soldiers Still in Hospital, 288
South Africa: School Medical Service, 515, 576; Trained Nurses' Association, 93, 394; News From, 286, 558
Southend: Corporation Health Committee, 120; Tuberculosis Hospital, 402
South London, New Hospital in, 596
South Wales, Another Hospital for, 29, 32
Southwark: Board of Guardians, 32, 42, 257, 354, 426, 521; Borough Council, 511
Spain, The Queen of, 440
Spencer, Mr. L. W. T., 217
Spiritual Side of Nursing, The, 64
Sportsmen and Charity, 287
Staffordshire Insurance Committee, 636
Stanley, Sir Arthur, 147, 161, 352
State Aid for Hospitals, 77, 162, May 15, vi, 211, 217, 289
State Medical Service, 425
State Registration for Nurses (*see also* College of Nursing and General Nursing Council), 26, 65, 162, 438, 553, 607, 625, 645
Stern, Sir Edward, 175
Still, Miss Lloyd, 510
Stopes, Dr. Marie, 352
Stralsund, Prisoners of War Library, 503
Streatham War Hospital Supply Depôt, 120
Streets, The Medical, 496
Suffolk County Nursing Association, 282
Suggestion to the Three Funds, A, 313
Suicide of a Nurse, 68
Summer School of Eugenics, 414
Supply of Doctors, The, 275
Surgical Instrument Makers' Association, 107
Survey of Hospitals, 199, 236, 373, 383
Swansea, Craig House, 647
Swedish Isolation Hospital, Life in a, Supp. April 3, 10, 17
Swift, Dame Sarah, 647
Syphilis (*see* Venereal Diseases)

Tata, Sir Ratan, The late, 162
Tax-Free Benefactions, 386, 426, 448, 476
Tea Drinking in India, 217
Telephone-Box, The Dangerous, 246, 385, 420
Tenby, St. John's Hospital, 32
Tennis Competition for Nurses, 626
Territorial Medical and Veterinary Services, 128
Tetanus: Incidence in the War, 422; Prevention of, 523
Theatrical Garden Party, April 10, vi, 288
Therapeutic Substances, The Control of, 105
Therapy, Fads and Fashions in, 549
Third London General Hospital, 377, 427, 440, 466, 556
Throat Irrigator, A Unique, Supp. July 10
Thromboangitis Obliterans, 83
Tied House in Surgical Practice, 61, 85, 182, 209
Timbril, Miss, 108
"Time and Tide," 215
*Times Woman's Supplement, The*, 366
Tod, Miss Winifred, 440
Tottenham District Nursing Association, 41
Trade Union Congress, 425
Trade Unions and Hospitals, 401, 409
Training of Nurses: Extended Term, 255; Recruiting for the Schools, 363
Transvaal: Trained Nurses' Association, 186; School Medical and Nursing Service, 515
Tropical Diseases, Medical Congress at Brisbane, 594
Treub, Dr. H., 53
TUBERCULOSIS: Hull School Children, 31; Sanatorium Readmissions, 31; Essex, 31; Notes, 36, 60, 526; Free Bedding, 108; Sanatorium Figures, 96; Treatment, 123; Congress at Leeds, 203; Outlying Sanatoria, 222; In the Printing Trade, 243; Proposed Sanatorium at Danbury, 336; Hospital for Southend, 403; New Sanatoriums in Wales, 425, 519; Backyard Beds, 428; Plain Speaking on Tuberculosis Matters, 446; Scheme for Advanced Cases, 488; Conference at Liverpool, 522; Salaries of Nurses, 555; Psychology, 574; Surgical Scheme, 600; Trips to Switzerland, 612; Sanatorium Patrol Officers, 618
"Twilight Sleep" Home, 626
Tynemouth Board of Guardians, 68
Typhus, 608, 623, 634, 638

Unemployment Insurance Bill, 299, 450, 537
Uniform System, Extension of the, 244
United Services Nurses' Club, 439
United States: National Civic Federation, 44; Public Health Committee, 45; Medical Ambassadors, 56; Cost of Clothing, 94; Red Cross, 94; Employment of Disabled, 98; Public Health Nursing, 556; Hospital Nomenclature, 628

Vaccination: The Value of, 52, 79, 128; Protection of Wounds, 406
V.A.D. (*see also* Red Cross): Work of London Detachment, 42; Club, 57, 330; Out-Patients, 107; Army, 118; Club, 175; District Nursing, 217; Meeting at Royal Automobile Club, 258; Cot in Memory of Workers, 306; Hertfordshire Nurses, 491; Metropolitan Asylums Board, 533
VENEREAL DISEASES (*see also* National Council): A Factor on Control, 28; In the Army, 212; Confidential Notification, 218; Prevention, 374, 411; Amongst Seamen, 504; Instruction in, 542; "Needs Must When—"
Ventilation, 633
Vertigo, 296
Victoria Cross Open for Women, 330
Victoria Nursing Association, 628
Vital Statistics, 530
Vitamine, A Stable, 86
Vivisection, 638
Voluntary Hospital Finance, 212, 338
Voluntary System, The, 326
Voluntary Insurance by Patients, 423, 431
Voluntaryism, A Testator and, 522

Wakley, Dr. James, 321
Walford, Mr. E. L., The Late, 612
Walsall, Waiting Lists, 336
Wandsworth Board of Guardians, 404, 512, 636
Ward, Mrs. Humphry, 17
War Certificate for Hospitals, 378
War Conditions in the Near East, 62
War Pensions Records, Supp. Sept. 18
War Library, The, 15
War Work, Inscribed Scroll for Hospitals, 464
Waterlow, Sir S. H., 320
Weil-Felix Reaction, The, 193
Welfare and Labour, 529
Welfare of Workers, 70
Welsh Ambulance Service, A, 543
Welsh Hospital, Netley, 29
Welsh School of Social Service, 560
West Ham Guardians, 554
West Riding County Council, 233
West Riding County Nursing Association, 41
Who Was Who, 278
Widening the Area of Hospital Support, Supp. May 1, 8
Wider Field, A, 314
Wilkins, Mr. S., 634
"Will to Health," The, 263
Willesden: Urban District Council, 218; Guardians, 636
Williams, David, 37
Williams, Dr. J. F., 478
Wills of Medical Men, April 10, vi, 238, August 28, vi, 612, Sept. 25, vi
Woman Committeeman, The, 477
Woman Worker's World, The, 470
Women and Nursing, 361
Women Doctors, 459, 478
Women Sanitary Inspectors, June 12, vi
Women War Workers, State Aid for, 90
Women's Holiday Fund, 284
Women's National Health Association, 234
Woolwich Nursery for Children, 67
Worcester, County Insurance Committee, 617
World Health, 506
Worker's Newspaper, The, 131
Workhouses, Children in, 643
Working Boys' Club, 604
Wounded Men Still in Hospital, 555

X-Rays, Some New Uses, 110

Y.M.C.A.: Demobilised Men, 303; Ducane Road Hospital, 480
York City Council, 81, 218
York, Duke of (*see* Prince Albert)
York Maternity Hospital, 282

PRINTED BY

SPOTTISWOODE, BALLANTYNE AND CO. LTD.

LONDON, COLCHESTER AND ETON

THE EDITOR'S LETTER-BOX—(*continued*).

### Scrapping the Trained Nurse.

### Gifts and Bequests.

Coventry and Warwickshire Hospital has been left £250 under the will of Mr. John Powers.

Emily Lady Dormer, of Bromley, Kent, has left £300 to St. Joseph's Hospice for the Dying.

Miss R. J. Horlock, of Clifton, Bristol, has left £1,000 to the Bristol Royal Hospital for Sick Women and Children for a "Mary Horlock Cot."

Ilkeston Hospital has been left £500 under the will of Mrs. Crompton, of Regent's Park.

Lady Tate, the widow of Sir Henry Tate, founder of the Tate Gallery, has left £1,000 each to Charing Cross Hospital, the Middlesex Hospital, and the Cancer Hospital; £500 to Brixton Nursing Association, and £3,000 to the British Home for Incurables, Streatham.

### The College of Nursing (Limited by Guarantee).

THE YORKSHIRE CENTRE AT LEEDS.

The annual meeting of the Yorkshire Centre will be held at Collinson's Café, Albion Street, Leeds, on Wednesday, April 21, at 7 p.m., when the committee and officers for the year will be elected. The following is a list of committee members who retire, but are eligible for re-election; other nominations for officers and members of committee must reach the Hon. Secretary not later than Saturday, April 10: *Officers: President and Local Representative*, Miss Innes, R.R.C.; *Hon. Treasurer*, Miss Edwards; *Hon. Secretary*, Miss Lindall. *Committee*: Mrs. Brothers (Leeds), Miss Barnes, R.R.R.C. (Leeds), Miss Horton (Harrogate), Miss Pressland (Wakefield), Miss Spann (Leeds), Miss Steele (York), Miss Tomlin, R.R.C. (Leeds), and Miss Whiffin, R.R.C. (Leeds). Only the votes recorded by members actually attending this meeting will be valid.

### The Book Market.

J. Wright & Sons: "Diseases of the Throat, Nose, and Ear." By W. G. Porter, M.B., etc. 3rd edition revised by A. Logan Turner, M.D. 12s. 6d. net.

John Murray: "Cunningham's Manual of Practical Anatomy." Vol. I. Sixth edition. Revised and edited by Arthur Robinson. 12s. 6d. net. "Syphilis in Childhood." By Leonard Finlay, M.D. 8s. 6d. net.

Methuen and Co., Ltd.: "Chemistry for Public Health Students." By E. Gabriel Jones, M.Sc., F.I.C. 6s. net.

William Heinemann: "Radiography in the Examination of the Liver, Gall, Bladder, and Bile Ducts." By Robert Knox, M.D. 7s. 6d. net. "Home Exercises for Spinal Curvature." By Richard Temberg, M.R.C.S. 6s. net.

Cassell and Co., Ltd.: "A Textbook of Gynæcological Surgery." By Comyns Berkeley and Victor Bonney. 42s. net.

Grant, Richards, Ltd.: "Broken Bridges." By Joseph Thorp. 5s. net.

### Parliament and the Professions.

### Miners' Nystagmus.

### Matrons' and Sisters' Appointments.

Freemasons' War Hospital.—Miss Florence Harrison has been appointed matron. She was trained at the West Suffolk General Hospital, where she was on the private nursing staff for some years. Subsequently she held the post of sister and night superintendent at the Ramsgate General Hospital, several posts at the Kent and Canterbury Hospital, and sister, as well as having charge of the nurses' home, at the Essex County Military Hospital, Colchester.

Wirral Union Infirmary, Clatterbridge, Bebington, near Birkenhead.—Miss Agnes Hollingworth and Miss Elizabeth Olive Kingdon have been appointed ward sisters. Miss Hollingworth was trained at Rochdale Infirmary, where she was later staff nurse and sister. She holds the certificate of the Central Midwives Board, and has done district nursing. Miss Kingdon was trained at Prescot Union Infirmary, where she was later charge nurse, and she has done private nursing in Manchester and Newcastle-on-Tyne.

Willesden Infirmary, Acton Lane, Willesden.—Miss Maud B. Johnson and Miss May Ireland have been appointed sisters. Miss Johnson was trained at the Dreadnought Hospital, Greenwich, and at the Hospital for Women, Soho, and she has held the posts of ward sister and night sister. Miss Ireland was trained at Willesden Infirmary, has held the post of staff nurse, and has done private nursing.

Victoria Hospital, Blackpool.—Miss H. Gould has been appointed staff nurse. She was trained at Halifax Royal Infirmary and at the Longton Accident Hospital, has been nurse in charge of the out-patient department, Kensington General Infirmary, senior sister at Pembroke Auxiliary Military Hospital, and sister at the Manchester Babies' Hospital, Levenshulme.

